# Effect of Text Message, Phone Call, and In-Person Appointment Reminders on Uptake of Repeat HIV Testing among Outpatients Screened for Acute HIV Infection in Kenya: A Randomized Controlled Trial

**DOI:** 10.1371/journal.pone.0153612

**Published:** 2016-04-14

**Authors:** Peter M. Mugo, Elizabeth W. Wahome, Evanson N. Gichuru, Grace M. Mwashigadi, Alexander N. Thiong’o, Henrieke A. B. Prins, Tobias F. Rinke de Wit, Susan M. Graham, Eduard J. Sanders

**Affiliations:** 1 Centre for Geographic Medicine Research – Coast (CGMR-C), Kenya Medical Research Institute, Kilifi, Kenya; 2 Department of Global Health, Academic Medical Center, University of Amsterdam, Amsterdam, the Netherlands; 3 Amsterdam Institute for Global Health and Development (AIGHD), Amsterdam, the Netherlands; 4 Departments of Medicine, Global Health, and Epidemiology, University of Washington, Seattle, United States of America; 5 Nuffield Department of Medicine, University of Oxford, Headington, United Kingdom; University of New South Wales, AUSTRALIA

## Abstract

**Background:**

Following HIV-1 acquisition, many individuals develop an acute retroviral syndrome and a majority seek care. Available antibody testing cannot detect an acute HIV infection, but repeat testing after 2–4 weeks may detect seroconversion. We assessed the effect of appointment reminders on attendance for repeat HIV testing.

**Methods:**

We enrolled, in a randomized controlled trial, 18–29 year old patients evaluated for acute HIV infection at five sites in Coastal Kenya (ClinicalTrials.gov NCT01876199). Participants were allocated 1:1 to either standard appointment (a dated appointment card) or enhanced appointment (a dated appointment card plus SMS and phone call reminders, or in-person reminders for participants without a phone). The primary outcome was visit attendance, i.e., the proportion of participants attending the repeat test visit. Factors associated with attendance were examined by bivariable and multivariable logistic regression.

**Principal Findings:**

Between April and July 2013, 410 participants were randomized. Attendance was 41% (85/207) for the standard group and 59% (117/199) for the enhanced group, for a relative risk of 1.4 [95% Confidence Interval, CI, 1.2–1.7].Higher attendance was independently associated with older age, study site, and report of transactional sex in past month. Lower attendance was associated with reporting multiple partners in the past two months.

**Conclusions:**

Appointment reminders through SMS, phone calls and in-person reminders increased the uptake of repeat HIV test by forty percent. This low-cost intervention could facilitate detection of acute HIV infections and uptake of recommended repeat testing.

**Trial Registration:**

Clinicaltrials.gov NCT01876199

## Introduction

HIV transmission remains high in Kenya with approximately 100,000 new infections annually [1]. Up to 40% of new HIV infections are estimated to stem from individuals in the acute stage (within one month of infection) or early stage (within 6 months of infection) [2]. The main factors for this high contribution include the very high viral loads during this period and continuing high risk sexual behaviour in the 6-month period following HIV-1 acquisition [3, 4] [5]. Not being aware of the infection may contribute to this continuing risk behaviour [4]. In the 2012 Kenya AIDS indicator survey, about half of all HIV-infected persons were unaware they were infected [6]. Diagnosing acute HIV infection (AHI) can facilitate counselling to reduce onward transmission as well as notification of recent sexual partners, who may also be at risk of HIV infection or have AHI or undiagnosed prevalent HIV infection [7–9]. However, strategies for AHI testing are currently lacking. Rapid point-of-care RNA or p24 antigen tests are not yet widely available in developing world settings [10, 11].

Two to four weeks following HIV-1 acquisition, many people develop a set of non-specific symptoms, commonly referred to as acute retroviral syndrome (ARS), and frequently seek urgent care [5, 12, 13]. As most participants seroconvert within 1–2 weeks following development of ARS, clinicians should invite participants for repeat antibody testing after 2–4 weeks if they suspect AHI [14]. Current guidelines recommend such re-testing for persons with discrepant rapid test results (i.e., one test positive and one negative) regardless of presence of symptoms [15, 16], but not those with ARS and negative for HIV antibodies [11, 17].

Few studies have assessed interventions aimed at increasing uptake of repeat HIV testing. With regard to routine HIV and sexually transmitted infection (STI) screening, three Australian studies found short message service (SMS) reminders to be effective in increasing repeat testing at 3–6 months [18–20], while a 2014 UK study found no effect of SMS reminders on repeat testing at 4 months [21]. In Kenya, there are few reports of similar studies. Among adult males undergoing circumcision in Kenya, SMS reminders increased attendance at the 7-day post-operative clinic visit [22]. A recent systematic review comprising studies in varying disease and clinical settings, mostly in the developed world, found that simple reminders that provided details of timing and location of appointments increased attendance and “should be sent to all participants in the absence of any clear contraindication” [23].

The objective of this study was to determine the effect of SMS, phone-call and in-person reminders on uptake of repeat HIV testing among outpatients evaluated for AHI in Coastal Kenya.

## Materials and Methods

### Ethics statement

The study protocol was approved by the ethical review committees at the Kenya Medical Research Institute (KEMRI), and the University of Oxford. All participants provided written informed consent prior to enrolment. The protocol was registered at https://clinicaltrials.gov/ct2/show/NCT01876199, registration number NCT01876199.

### Trial setting and study population

This trial was nested within a larger study of targeted evaluation for AHI, see S1 Protocol and S1 Text [24]. In brief, the AHI study enrolled participants 18–29 years old seeking care at five health facilities and five community pharmacies in Coastal Kenya. Pharmacy clients were referred to any of the participating health facilities (study sites) based on their preference. The trial was coordinated from a KEMRI research clinic in the study area.

### Procedures

See S1 Protocol and S1 Text [24] for detailed study procedures. Briefly, enrolled participants were tested for HIV at the care-seeking visit using two rapid antibody tests in parallel. For seronegative patients, the counsellor explained that repeat testing was needed to ensure that HIV infection was not the cause of the patient’s symptoms. A blood sample was taken for laboratory-based p24 antigen and pooled RNA testing. Results of the p24 antigen test were available within a day of sample collection, while pooled RNA testing was done at the end of the study. Locator information was collected from all participants, including mobile phone number and home or workplace address depending on participant’s preferred contact location. Phone numbers were dialed at the time of collection to confirm that they were valid. As streets are not numbered in this setting, the address information included the nearest landmark plus a sketch map to the house. All participants who tested HIV antibody negative were invited for repeat rapid antibody testing two weeks after the initial test. Participants with a positive p24 antigen test result were contacted immediately and invited to the KEMRI clinic for further testing and counseling; participants with a negative p24 antigen test were given this result at their 2-week follow-up visit.

### Randomization

Randomization was introduced following a one-month lead-in period in the AHI study. Participants were randomized to either standard appointment or enhanced appointment on a 1:1 ratio using the sealed opaque envelope method. Envelopes were prepared by a data manager not involved in screening, enrolment and follow-up of participants. Randomization was stratified by study site. Un-numbered envelopes were supplied to study sites in shuffled batches of twenty, 10 for standard appointment and 10 for enhanced appointment. When fewer than 6 envelopes were remaining at a study site, a new set of 20 envelopes was supplied. After enrolment, HIV testing, and all other enrolment visit procedures, the attending clinician asked the participant to pick one envelope at random. Neither participants nor study staff were blinded to the assigned group, as blinding was not feasible given the nature of the intervention.

### Interventions

Standard appointment consisted of instructions to come back to the clinic on a specific date two weeks after the enrolment visit, plus an appointment card with the appointment date and participant number written on it. Standard appointments were issued by the attending clinician. Enhanced appointment comprised of the standard appointment plus phone reminders (SMS and phone call) for participants who owned a cell phone or in-person reminders (a home or workplace visit by a fieldworker) for participants without a phone.

In-person reminders for those without a phone were delivered two to four days before the scheduled appointment date and were not repeated if the appointment was missed. Phone reminders were delivered using a basic feature phone by the same fieldworker delivering in-person reminders. Participants received a pre-appointment SMS the day before the scheduled appointment date plus missed-appointment reminders escalated as follows: a second SMS the day after the scheduled date, a phone call on the third day, and an in-person reminder (physical tracing) four to seven days after the scheduled date for those who could not be reached on phone. For purposes of determining the need for reminder escalation, visit attendance was confirmed from the participant file through daily visits or phone calls to the attending clinicians.

SMS text messages were in Kiswahili and were identical for all participants. Participants were not required to reply to the SMS and no incentives were given with regard to reminders. The first SMS read: “Please remember to go for your clinic appointment tomorrow. Call this number if you need more information”, while the second SMS read: “You missed your clinic appointment yesterday. Please report to the clinic as soon as possible.” For confidentiality reasons, participants using a friend’s phone contact were called but not sent SMS if they preferred phone reminders to in-person reminders. Phone reminder attempts were recorded in an Excel^®^ spreadsheet and confirmed against a printed log from the mobile operator.

### Outcomes

The primary outcome measure was visit attendance defined as the proportion of participants attending their follow-up visit for repeat HIV testing within two weeks of the scheduled date. Since the aim was not to measure timelines of attendance, we assumed that the effect of reminders is the same whether delivered before the appointed date or after a missed appointment, hence the outcome could be compared across in-person and phone reminders subgroups despite this asymmetry in the timing of reminders.

### Statistical methods

The sample size used was based on the objectives of the primary study, specifically the objective to accrue 12 AHI cases (see S1 Protocol). This sample size (n = 412) provides 98% power, for a 2-sided significance level of 0.05, to detect a difference in visit attendance across randomization groups assuming 50% visit attendance in the intervention group vs 30% in the control group. A sample size half as big would give 90% power to detect a similar difference.

Analyses were done in Stata^®^ version 13 (StataCorp, College Station, Texas). The primary analysis of the intervention effect followed the intention-to-treat principle, in which participants were included in the group to which they were randomized, irrespective of whether they received the allocated intervention. A secondary as-treated analysis was performed in which participants were grouped according to the intervention they received (“treatment” group). Relative risks were calculated along with 95% confidence intervals (CIs). Interactions between baseline characteristics and randomization group were assessed using the Mantel-Haenszel method, with p<0.05 from the test of homogeneity indicating significant interactions.

In other analyses, visit attendance was compared across phone reminders and in-person reminders sub-groups, and the contribution of individual components of phone reminders to the intervention effect was assessed by calculating the proportion attending at each stage of the reminder escalation. All participants who had a valid telephone number at enrolment, including those who were subsequently unreachable, were categorized in the phone reminders sub-group. This analytical approach is in line with the intention-to-treat concept, though it is important to note that this was not a randomized grouping.

To identify factors independently associated with visit attendance, we conducted bivariable and multivariable logistic regression. Treatment group was included *a priori*. Other variables with Wald p<0.10 from bivariable analyses were included in an initial multivariable model. Variables not originally selected from the bivariable analyses were then added back into the initial model, one at a time, and any with significant association (two-sided p-value<0.05) included to make the full model. Factors with p<0.05 in the full model were considered to be statistically significant.

## Results

### Flow of participants

**Fig 1:**
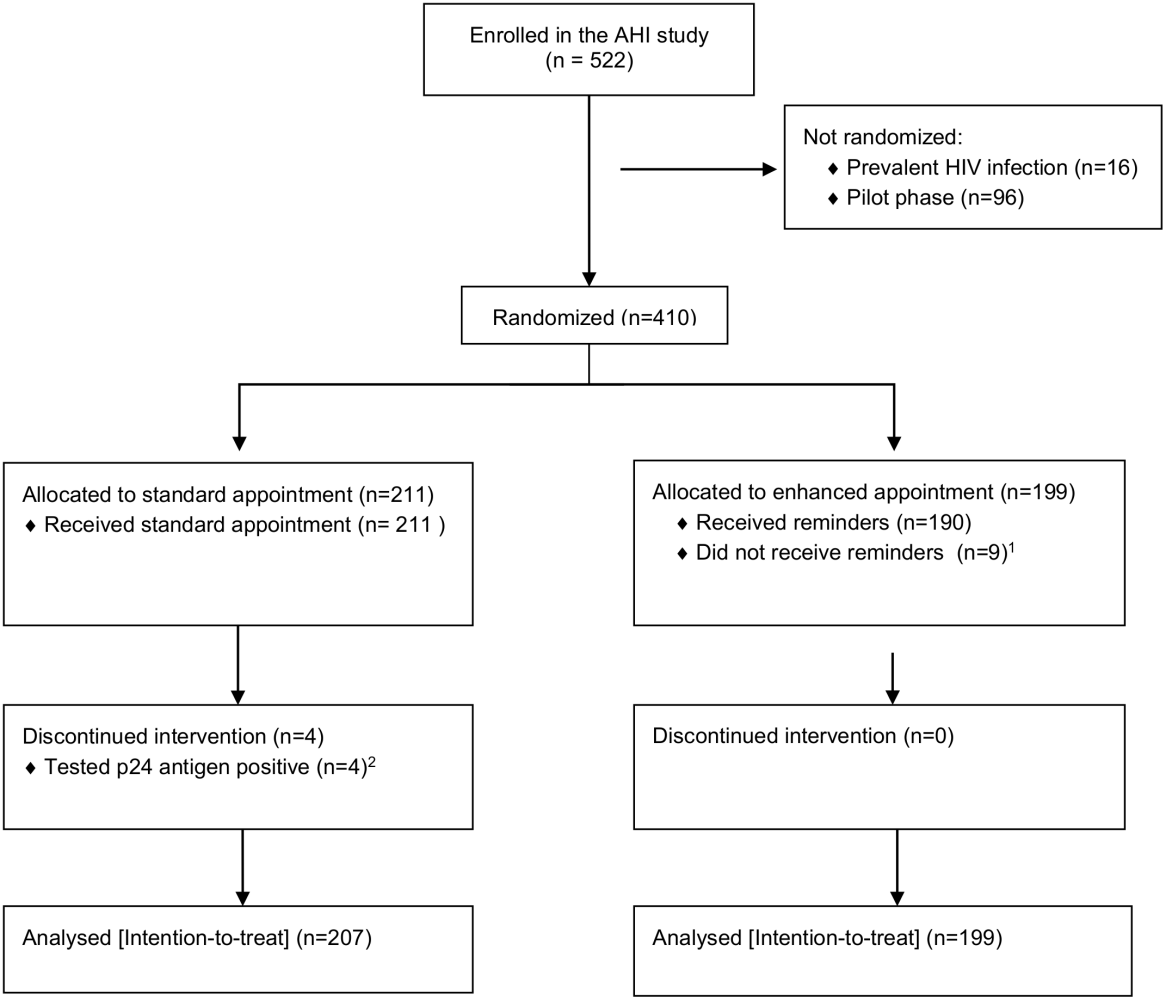
Flow of participants. ^1^Reminders were not sent because locator forms were missing. ^2^Participants who tested p24 antigen positive were recalled to the clinic immediately, hence not included in the analysis of intervention effect.

Between April and July 2013, 410 participants were randomized into the trial (Fig 1). Of 199 participants allocated to enhanced appointment, 150 had a cell phone and received SMS and phone-call reminders, 40 did not have a cell phone and received in-person reminders, while nine did not receive any reminders because locator forms were missing at the time of sending reminders. The nine locator forms were completed at the follow-up visit.

### Baseline characteristics of participants

Table 1 presents the demographic and clinical characteristics of trial participants. Overall, majority of trial participants were female (65%), had primary or secondary education (80%), were single (58%), owned a cell phone (79%), and had tested for HIV before (75%). All participants were young (mean age 23 years) as per the design of the primary study. Randomization groups were well balanced on all characteristics except for minor imbalances on two variables, initial point of care-seeking and pregnancy, possibly resulting from the relatively large block size.

**Table 1:** Baseline characteristics of participants.

Characteristic	Standard appointment group N (%)	Enhanced appointment group N (%)	Both groups combined N (%)
**Number of participants:**	211	199	410
**Gender:**			
Male	77 (36%)	67 (34%)	144 (35%)
Female	134 (64%)	132 (66%)	266 (65%)
**Age:**			
18–24 years	140 (66%)	120 (60%)	260 (63%)
25–29 years	71 (34%)	79 (40%)	150 (37%)
**Level of education:**			
None	11 (5%)	11 (6%)	22 (5%)
Primary	82 (39%)	73 (37%)	155 (38%)
Secondary	91 (43%)	82 (41%)	173 (42%)
Tertiary	27 (13%)	33 (17%)	60 (15%)
**Marital status:**			
Single	127 (60%)	112 (56%)	239 (58%)
Married	80 (38%)	84 (37%)	164 (40%)
Separated/divorced	4 (2%)	3 (2%)	7 (2%)
**Source of income:**			
No income	22 (10%)	15 (8%)	37 (9%)
Family	84 (40%)	77 (39%)	161(39%)
Employed	59 (28%)	57 (29%)	116 (28%)
Self-employed	46 (22%)	50 (25%)	96 (23%)
**Phone contact**^1^**:**			
Own cell phone	169 (80%)	155 (78%)	324 (79%)
Other cell phone^1^	29 (14%)	26 (13%)	55 (13%)
No phone contact	13 (6%)	18 (9%)	31 (8%)
**Treatment sought**^2^**:**			
Fever (yes)	102 (48%)	96 (48%)	198 (48%)
Diarrhea (yes)	33 (16%)	37 (19%)	70 (17%)
STI symptoms (yes)	86 (41%)	76 (38%)	162 (40%)
Body pains (yes)	151 (72%)	141 (71%)	292 (71%)
**Initial point of care seeking:**			
Pharmacy	39 (18%)	51 (26%)	90 (22%)
Health facility	172 (82%)	148 (74%)	320 (78%)
**Enrolling study site**^3^**:**			
A	95 (45%)	92 (47%)	187 (46%)
B	36 (17%)	35 (17%)	71(17%)
C	28 (13%)	27 (14%)	55 (13%)
D	37 (17%)	31 (16%)	68 (17%)
E	15 (7%)	14 (7%)	29 (7%)
**Too sick to do normal activities**	35 (17%)	30 (15%)	65 (16%)
**Pregnant (females only)**	12 (9%)	3 (2%)	15 (6%)
**>1 sex partner in past 2 months**	31 (15%)	31 (16%)	62 (15%)
**Transactional sex in past 4 weeks**	8 (4%)	7 (4%)	15 (4%)
**Ever been tested for HIV**	155 (73%)	154 (77%)	309 (75%)

^1^This includes cell phones belonging to the partner (n = 23), family member (n = 21), friend (n = 7) or neighbor (n = 4) to the trial participant.

^2^ 279 participants sought treatment for more than one symptom (standard 147, enhanced 132); including 246 with two symptoms (standard 133, enhanced 113), and 33 with three symptoms (standard 14, enhanced 19).

^3^ Sites A and C are government health facilities, while sites B, D and E are private health facilities.

### Intervention effect

In the intention-to-treat analysis (ITT), visit attendance was 41% (85/207) for the standard group and 59% (117/199) for the enhanced group, for a relative risk of 1.4 [95% Confidence Interval, CI, 1.2–1.7]. In the as-treated analysis, nine participants allocated to enhanced appointment but not sent reminders were re-categorized to the standard “treatment” group. Attendance calculated using this as-treated approach was 42% (91/216) for those who received the standard appointment and 58% (111/190) for those who received appointment reminders, for a relative risk of 1.4 [95% CI, 1.1–1.7]. No interactions were detected. The absolute improvement in attendance, by the ITT approach, was 18% [95% CI 8%-27%] and the number needed to “treat” (NNT) for one additional patient to attend was 6 [95% CI 4–12].

**Fig 2:**
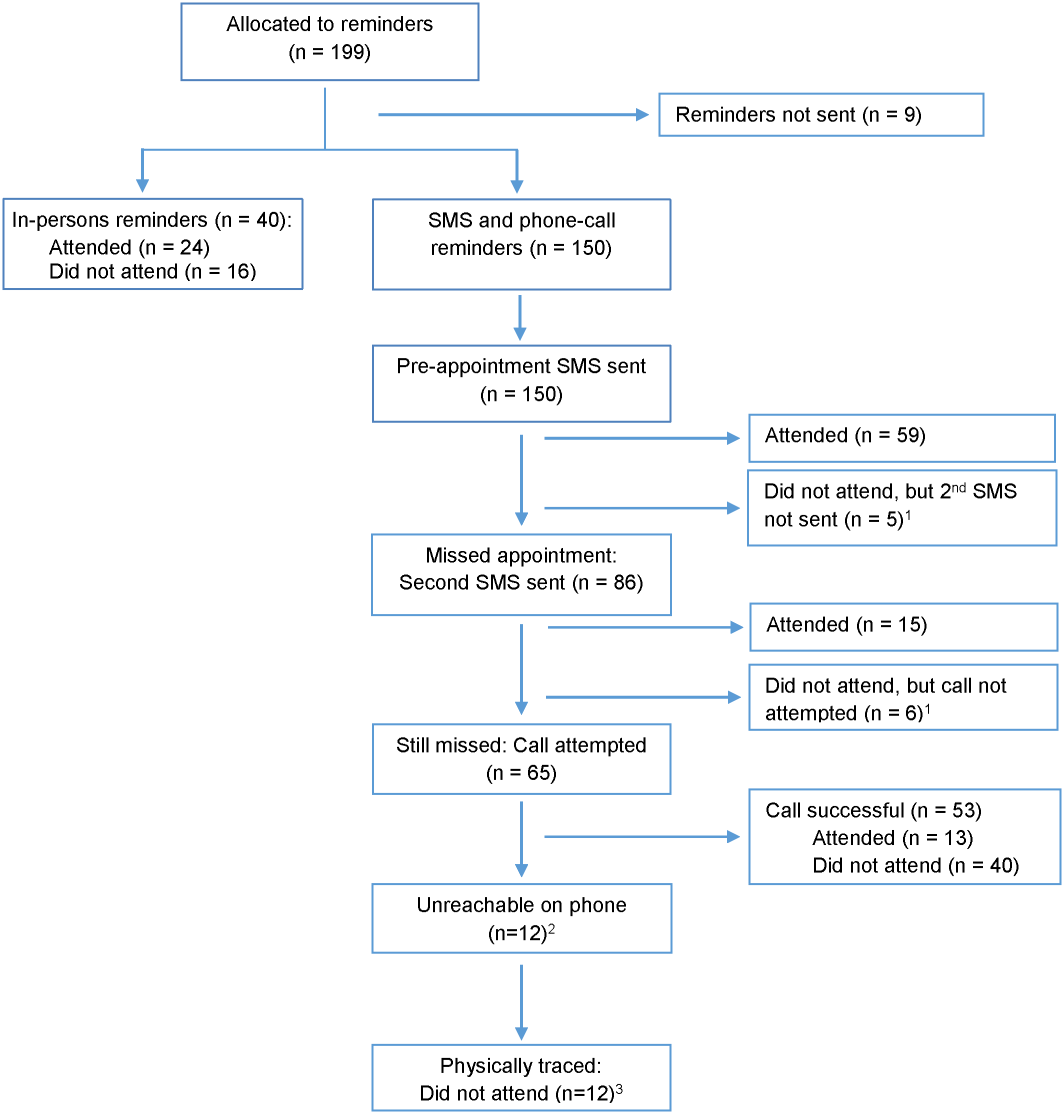
Delivery and outcome of appointment reminders. ^1^Due to delays in communication from study sites, follow-up reminders were not sent for five participants after the first SMS and for six participants after the second SMS. ^2^Twelve participants who had provided a valid mobile at enrolment were subsequently unreachable at the follow-up visit, perhaps due to lost mobile phone or changed numbers. ^3^Three participants could not be found at the address they had given on the locator form, but we could not determine if they had given incorrect information or had moved.

Fig 2 shows the delivery and outcome of reminders in the enhanced appointment group. Visit attendance was 60% (24/40) for the in-person reminders sub-group and 58% (87/150) for the phone reminders sub-group (p = 0.8). In the phone reminders sub-group, visit attendance at each stage of the reminders escalation was as follows: 39% (59/150) after the pre-appointment SMS was sent, 49% (74/150) after the second SMS was sent, and 58% (87/150) after phone calls were made. This outcome remained unchanged after physical tracing of those who were unreachable on phone.

As we have previously reported [24], all the AHI cases identified in the study were detected through p24 antigen testing at the initial care seeking visit. No additional HIV infections were detected in participants who took the repeat test. Pooled RNA testing of samples from participants who did not take a repeat test revealed no additional infections. See S1 Text.

### Factors associated with uptake of repeat HIV testing

Table 2 presents regression analysis results, showing factors associated with visit attendance for repeat HIV testing. Higher attendance was significantly associated with receiving appointment reminders, older age, study site, and transactional sex in past month. Lower attendance was significantly associated with reporting multiple (>1) sex partners in past two months.

**Table 2:** Factors associated with visit attendance for repeat HIV testing.

Characteristic	N Expected at follow-up visit	N Attending follow-up visit (% of expected)	Bivariable analysis		Multivariable analysis (Full model)	
			Odds ratio [95% Confidence interval]	Wald P value	Adjusted Odds ratio [95% Confidence interval]	Wald P value
**Intervention received:**						
Standard appointment	216	85 (42%)	Ref	Ref	Ref	Ref
Standard appointmentplus reminders	190	117 (58%)	1.9 [1.3–2.9]	0.001	2.0 [1.3–3.0]	0.001
**Gender:**						
Male	144	70 (48%)	Ref	Ref	—	—
Female	262	132 (50%)	1.1 [0.7–1.6]	0.7	—	—
**Age:**						
18–24 years	258	118 (46%)	Ref	Ref	Ref	Ref
25–29 years	148	84 (57%)	1.5 [1.0–2.3]	0.03	1.7 [1.1–2.6]	0.02
**Level of education:**						
None	22	12 (55%)	1.5 [0.6–3.7]	0.4	1.3 [0.5–3.3]	0.6
Primary	152	67 (44%)	Ref	Ref	Ref	Ref
Secondary	172	93 (54%)	1.5 [1.0–2.3]	0.07	1.5 [0.9–2.5]	0.1
Tertiary	60	30 (50%)	1.3 [0.7–2.3]	0.4	1.3 [0.7–2.5]	0.5
**Marital status:**						
Single	237	117 (49%)	1.0 [0.7–1.5]	1.0	—	—
Married	163	80 (50%)	Ref	Ref	—	—
Separated/divorced	6	5 (83%)	5.2 [0.6–45]	0.1	—	—
**Source of income:**						
No income	37	19 (51%)	1.3 [0.7–2.9]	0.5	—	—
Family	160	79 (49%)	1.2 [0.7–2.0]	0.4	—	—
Employed	114	62 (54%)	1.5 [0.9–2.6]	0.1		
Self-employed	95	42 (44%)	Ref	Ref	—	—
**Phone contact**						
Own cell phone	320	161 (50%)	1.2 [0.6–2.6]	0.7	—	—
Other cell phone	55	27 (49%)	1.2 [0.5–2.8]	0.6	—	—
No phone contact	31	14 (45%)	Ref	Ref	Ref	Ref
**Treatment sought:**						
Fever						
No	210	114 (54%)	Ref	Ref	Ref	Ref
Yes	196	96 (49%)	0.7 [0.5–1.0]	0.06	0.7 [0.4–1.1]	0.09
Diarrhea						
No	337	163 (48%)	Ref	Ref	—	—
Yes	69	37 (53%)	1.4 [0.8–2.3]	0.2	—	—
STI symptoms						
No	244	118 (48%)	Ref	Ref	—	—
Yes	162	76 (47%)	1.1 [0.8–1.2]	0.5	—	—
Body pains						
No	117	61 (52%)	Ref	Ref	—	—
Yes	289	141 (49%)	0.9 [0.6–1.3]	0.5	—	—
**Initial point of care seeking:**						
Pharmacy	89	42 (47%)	Ref	Ref	—	—
Health facility	317	160 (50%)	1.1 [0.6–1.3]	0.6	—	—
**Enrolling study site:**						
A	186	97 (52%)	6.9 [2.3–20.3]	0.001	6.8 [2.1–21.7]	0.001
B	70	29 (41%)	4.4 [1.4–14.1]	0.01	4.1 [1.2–13.6]	0.02
C	54	27 (50%)	6.2 [1.9–20.4]	0.002	7.1 [2.1–24.9]	0.002
D	67	45 (67%)	12.8 [4.0–41.3]	<0.001	10.7 [3.2–36.2]	<0.001
E	29	4 (14%)	Ref	Ref	Ref	Ref
**Too sick to do normal activities**						
No	343	170 (50%)	Ref	Ref	—	—
Yes	63	32 (51%)	1.1 [0.6–1.8]	0.9	—	—
**Pregnant (females only)**						
No	246	127 (52%)	Ref	Ref	—	—
Yes	15	6 (40%)	0.6 [0.2–1.8]	0.4	—	—
**>1 sex partner in past 2 months**						
No	345	174 (50%)	Ref	Ref	Ref	Ref
Yes	61	28 (46%)	0.8 [0.5–1.4]	0.5	0.5 [0.2–1.0]	0.04
**Transactional sex in past 4 weeks**						
No	391	191 (49%)	Ref	Ref	Ref	Ref
Yes	15	11 (73%)	2.9 [1.0–9.1]	0.07	4.9 [1.2–19.6]	0.03
**Ever tested for HIV before**						
No	101	42 (42%)	Ref	Ref	Ref	Ref
Yes	305	160 (52%)	1.6 [1.0–2.4]	0.06	1.5 [0.9–2.4]	0.1

Except for three variables (>1 sex partner in past 2 months, transactional sex in past 4 weeks and ever tested for HIV before), multivariable modelling confirmed bivariable modelling, with minor changes in odds ratios and p-values. Having tested for HIV before was marginally associated with higher attendance in the bivariable model, but not in the multivariable model.

## Discussion

In this randomized trial among patients evaluated for acute HIV infection in Coastal Kenya, we found that appointment reminders through SMS, phone calls and in-person reminders increased the uptake of repeat HIV test 2–4 weeks after the initial test by forty percent.

To our knowledge, this was the first randomized trial to evaluate a reminder intervention aimed at increasing uptake of repeat HIV testing in a developing country setting. Our findings are consistent with most previous studies that evaluated effect of SMS reminders and phone calls on attendance to clinic visits for HIV/STI screening [18–20] and for other medical services [22, 25–28]. Overall, reminders seem to be more effective where baseline rates of attendance are low and in situations where a longer time has elapsed since the last clinic visit (roughly: relative risk 1.9–4.6 for studies with follow-up visits at three months or more vs. relative risk 1.09–1.9 for studies with follow-up visits after seven days to two months).

Our study included a component of in-person reminders for those without mobile phones; the effect of in-person reminders was similar to phone reminders. In-person contacts, also referred to as physical tracing, are more labour intensive and may be more costly than phone reminders, particularly where phone reminders do not involve use of expensive software. This additional cost is worthwhile, since the proportion of people without phones remains sizeable in developing country settings, even in fairly urbanized areas like the one in which the study was conducted; 21% of patients in our sample did not have phones. Our experience in this study suggests that patients in these settings are able and willing to provide accurate address information.

Our study provides evidence of the utility of follow-up phone calls for participants who don’t attend after being sent SMS reminders. This effect may indicate failure to read SMS reminders by some participants or may reflect the more personal nature of a phone call compared to SMS, particularly because our text messages were not personalized. On the other hand, for participants who provided a valid phone contact but were subsequently unreachable, physical tracing had no additional benefit. Being unreachable on phone may indicate unwillingness or inability to attend the clinic visit or to be contacted further.

While all the AHI cases in our study were identified by p24 antigen testing at the enrolment visit, repeat antibody testing after 2–4 weeks could help diagnose such cases in settings where p24 antigen or RNA assays are not available [29, 30]. Our reminder intervention could also be applicable to repeat HIV testing beyond AHI diagnosis or to any other condition requiring a follow-up clinic visit. The current Kenyan guidelines for HIV testing and counselling recommend retesting after two weeks for individuals with discrepant rapid test results; after one month for those with symptoms of STI or tuberculosis; after one month and three months for those with a specific exposure in the past 72 hours; after three months for individuals with a specific incident of exposure in the past three months; and every year for other HIV negative individuals with ongoing risk of infection [15, 16].

We found that younger patients and those reporting multiple sex partners were less likely to attend the repeat test visit. Younger individuals may view themselves as being at a lower risk of HIV infection while individuals reporting multiple sex partners may be hesitant to retest fearing a positive result. These groups may therefore require special attention during implementation of interventions for repeat HIV testing, particularly because they also have a high HIV acquisition risk [6, 31]. Study site had a strong association with visit attendance, driven by very low attendance at one clinic. This clinic had the smallest premises, number of participants and number of staff. Participants in this clinic were seen mainly by nurses, while in the other clinics participants were seen mainly by clinical officers. While these differences may explain the difference in visit attendance, other unmeasured factors may have played a greater role.

Our study had some limitations. First, we included only young adults aged 18–29 years as required by the AHI screening algorithm, hence the results may not be generalizable to older patients. Second, we did not collect data some factors that could impact visit attendance, such as distance to the clinic, hence some of the associations we identified may be the result of residual confounding. Third, participants and study staff were not blinded to the assigned groups, as blinding was not feasible given the nature of the intervention.

In conclusion, we found that appointment reminders through SMS, phone calls and in-person contacts increased the uptake of repeat HIV test by forty percent. This low-cost intervention could facilitate the early detection of HIV infections and uptake of recommended repeat HIV testing.

## Supporting Information

S1 CONSORT Checklist CONSORT checklist. (DOCX)

S1 Dataset (CSV)

S1 Protocol (PDF)

S1 Text Primary paper. (PDF)
