# Supplementary material for: Effect of Text Message, Phone Call, and In-Person Appointment Reminders on Uptake of Repeat HIV Testing among Outpatients Screened for Acute HIV Infection in Kenya: A Randomized Controlled Trial
Source: PLoS One. 2016 Apr 14;11(4):e0153612. doi: 10.1371/journal.pone.0153612 (PMC4831710; doi:10.1371/journal.pone.0153612)
Supplement: S1 Protocol — (PDF) [file pone.0153612.s003.pdf]

1) (Title): Targeted Screening of At-Risk Adults for Acute HIV-1 Infection

2) Investigators

Dr. Eduard Sanders (PI)

Peter Mugo (Co-PI)

Dr. Henrieke Prins

Alexander Thiong'o

Elizabeth Wahome

Haile Selassie Okuku

Elise van der Elst

Dr. Adrian Smith[1]

Dr. Anisa Omar[2]

Dr. Susan Graham

Affiliations of Non-KEMRI investigators

1). University of Oxford, UK

2). Provincial Medical Office, Mombasa. Ministry of Public Health and Sanitation

3) Abstract

It is estimated that up to 40% of HIV-1 transmission is attributable to sexual contact with individuals with acute HIV-1 infection (AHI), as high virus loads among patients who recently acquired HIV-1 and their continued sexual risk behaviour before diagnosis are major determinants of transmission. Patients who acquire HIV-1 frequently have symptoms prompting them to seek treatment from pharmacies or health care facilities. Unfortunately, pharmacists and clinical staff often neglect HIV-1 testing for such cases. Even if testing is offered, rapid HIV-1 tests will usually be negative or discordant. Point-of-care tests for HIV-1 RNA are not yet available. In this prospective study, we will use a risk score algorithm to identify approximately 600 adult patients seeking urgent health care for symptoms compatible with AHI or a sexually transmitted disease (STD) at pharmacies or health care facilities in Mtwapa and Shanzu. We will evaluate HIV-1 status in all eligible participants using rapid HIV-1 tests to diagnose prevalent HIV-1 infection and both a p24antigen test at the time of presentation and convalescent rapid HIV-1 tests obtained 2-4 weeks following presentation to diagnose AHI. We anticipate that approximately 480 participants will be HIV-1-seronegative or serodiscordant at presentation, assuming an HIV-1 prevalence of approximately 20% in this select high-risk population. Among

APPROVED

07 JAN 2013

all patients evaluated for AHI, we will conduct a pilot randomised controlled trial (RCT) to assess the efficacy of intensified follow-up vs. standard follow-up appointments to achieve completion of the testing algorithm. All patients with AHI (n=12, estimated), and all patients with prevalent HIV-1 infection who are not linked to care (n=100, estimated) will be offered risk reduction counselling and enrolment into comprehensive care at available care facilities in the area.

4) Introduction

Targeting individuals for HIV-1 testing and HIV prevention interventions in Kenya is challenged by two findings: (1) most (84%) HIV-1 infected adults in a recent National survey were unaware of their HIV-1 infection status, as 56% had never tested and 28% reported their last HIV-1 test was negative;[1] and (2) up to 40% of HIV-1 transmission is attributable to sexual contact with individuals with acute HIV-1(6 weeks after infection) or early HIV-1 infection (6 months after HIV-1 infection).[2, 3] Very high virus loads among persons who recently acquired HIV-1 and their continued sexual risk behaviour before diagnosis are major determinants of transmission.[4] Although National strategies recommend point-of-care HIV-1 testing for all adults accessing care (e.g. PMTCT), patients who seek health care at pharmacies are not targeted for HIV-1 testing to our knowledge. Among health facilities in the proposed study setting, our preliminary work suggests a low level of HIV-1 testing targeted at patients presenting with symptoms suggestive of AHI.

Adults who acquire HIV-1 frequently seek health care for symptoms.[5, 6 7] While some patients with acute HIV-1 infection (AHI) remain asymptomatic, the majority experience an acute illness approximately 2 weeks following infection.[8, 9] Common symptoms of AHI include fevers, joint and muscle pains, headache, fatigue and rash; a minority of patients have a typical mononucleosis-like illness with fever, sore throat, and oral ulcers.[10] Identification of patients with AHI represents an important opportunity to limit onward HIV-1 transmission if such patients can be engaged in care and offered risk reduction counselling.[11]

Diagnosing patients with AHI remains challenging in resource-limited settings, in part due to a lack of low-cost, point-of-care tests for nucleic acid testing.[11] When patients who acquire HIV-1 first present for urgent health care, rapid tests for HIV-1 antibodies will be negative or discordant,[12] but p24 antigen may be detected.[13] Although older p24 antigen assays were relatively insensitive, new assays have been developed with increased sensitivity, lower

APPROVED
07 JAN 2013

cost, and simpler technical requirements.[14] For patients in whom p24 antigenemia is not detected, repeat HIV-1 testing with readily available rapid tests will identify seroconversion in most acutely infected individuals 2-4 weeks following symptom onset.[8 15] While such an approach will miss individuals who do not seek health care, repeat rapid testing represents an approach that is currently feasible in resource-limited settings.

From: McMichael AJ, et al, Nature Reviews Immunology Jan 2010; 10: 11-23. Figure b depicts onset of symptoms, approximately 2 weeks following HIV-1 acquisition. Figure a (next page) depicts that p24ag and rapid HIV-1 tests may be detected as early as 14 and 20 days post acquisition, respectively.

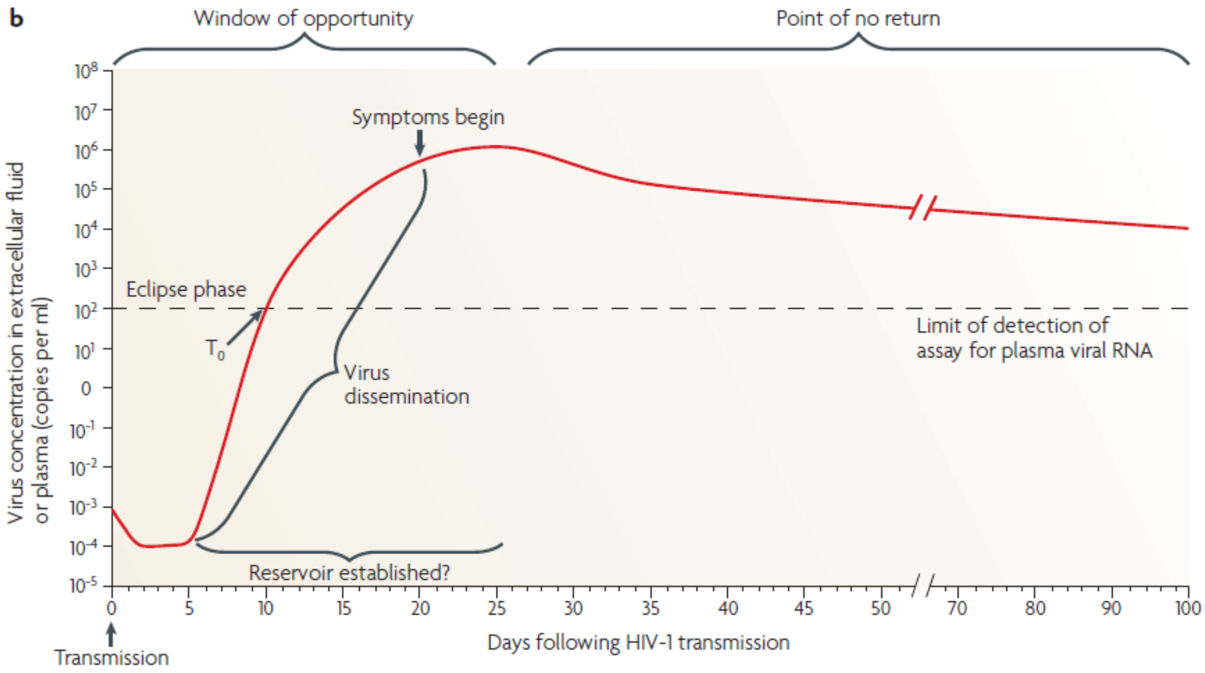

APPROVED
07 JAN 2013

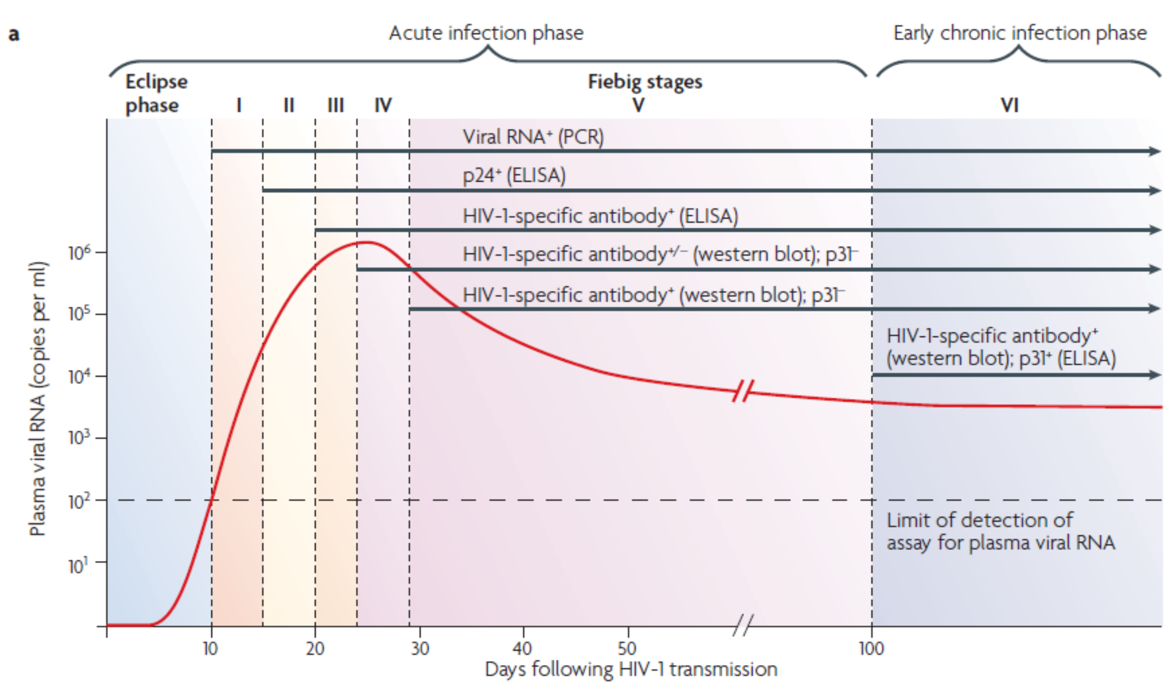

A recent study in South Africa using HIV-1 PCR testing showed that 6 (0.67%) of a sample of 902 HIV-1-seronegative at-risk adults presenting to VCT or STD services at a public health clinic were positive for HIV-1 RNA when tested. Sixty percent of this study population received HIV-1 test results and post-test counselling, including all 6 patients with documented AHI. Text message reminders and phone calls were employed to encourage patients to return for results, and patients with AHI were also visited at home.[16] In another study based in an outpatient department in Durban, South Africa with a very high HIV-1 prevalence (48%), pooled serum HIV-1 RNA screening was used to determine that 1.1% of all outpatients with negative or discordant rapid HIV-1 test results had AHI.[13] In Lilongwe, Malawi, in a prospective evaluation of methods to detect AHI in STD patients using a combination of rapid tests, p24antigen testing, and pooled RNA testing, approximately 90% of the 21 AHI cases detected were identified by discordant rapid results or p24antigen testing, without further need for RNA testing.[17]

Little information is available about health care seeking prior to HIV-1 seroconversion among adults in Africa. Among 44% of 103 women who seroconverted in Mombasa, the acute illness was severe enough to prevent them from working, but information on health care seeking was not available.[18] Studies in Uganda and Mozambique showed that 1%-3% of adults who sought care for suspected malaria actually had acute or early HIV infection,[19 20] with higher proportions in areas with higher HIV-1 prevalence. We have reported that 50 of 72 (69%) HIV-1-seroconverters identified in our prospective cohort study in Mtwapa and Kilifi had sought

APPROVED
07 JAN 2013

urgent health care for symptoms prior to seroconversion, and 29 of these individuals (40%) were incorrectly assumed to have malaria.

Risk score algorithms combining risk behaviour, selected symptoms, sexually transmitted diseases (STD) and discordant HIV-1 rapid test results may potentially be helpful in identifying patients with AHI.[12] As regular screening for AHI may have a low (<1%) yield in any patient screened, targeted screening will be more cost-effective if fewer patients will be screened for each case of AHI identified. A systematic review of 57 HIV-1 incidence studies in sub-Saharan Africa over the period 1987-2008 showed that young people (under <25 years of age), individuals in concurrent or multiple partnerships, and individuals with a current or recent sexually transmitted infection were consistently identified as being at risk for HIV-1 infection. [21]

A risk score algorithm developed by Powers et al. at an STD clinic in Malawi included reporting more than one sexual partner in the prior 2 months, fever, body ache, diarrhoea, genital ulcer disease, and rapid test results. Corresponding predictor scores to identify patients with AHI were 1 for fever, body ache, and more than one partner; 2 for diarrhoea and genital ulcer disease; and 4 for discordant rapid tests. A risk score of 2 or greater was 95% sensitive and 60% specific in detecting AHI.[12]

Upon evaluating the Powers' risk score algorithm among 683 men (including 448 (65%) MSM) participating in the high-risk cohorts in Kilifi and Mtwapa, the sensitivity was 81%, specificity 72%, and positive predictive value (PPV) 2.8% to identify AHI.[22] Using a similar methodology to Powers et al., we developed a cohort-derived risk score algorithm. Independent predictors for HIV-1 acquisition and corresponding predictor scores were: 1 for fever, STD (defined as diagnosed urethritis or proctitis), diarrhoea, or unprotected sex in the previous week; 2 for having taken treatment for "malaria" or a report of any anal sex in the past month; and 4 for discordant HIV-tests.[22] This score was 83.8% sensitive, 59.6% specific and had a PPV of 2.0% to identify AHI. A simplified algorithm, however, including the presence of either fever or an STD (i.e. urethritis or proctitis) and age <30 years was 74% sensitive, 76% specific, and had a PPV of 3.0% to identify AHI in high-risk men in Kilifi.[22 23] Of note, 25% of all participating men met the criteria of the simplified algorithm. Previously, we documented that median age (and interquartile range, IQR) for HIV-1 seroconversion among men and women was 24 (IQR; 22-28) and 25 (IQR: 23-27) years, respectively.[23]

APPROVED
07 JAN 2013

The figure below shows ROC curves for the risk score algorithms discussed. Both the Powers algorithm and the cohort-derived algorithm had similar good performance, while the simplified algorithm had modest performance, when evaluated in high-risk men followed for HIV-1 acquisition in Mtwapa and Kilifi.

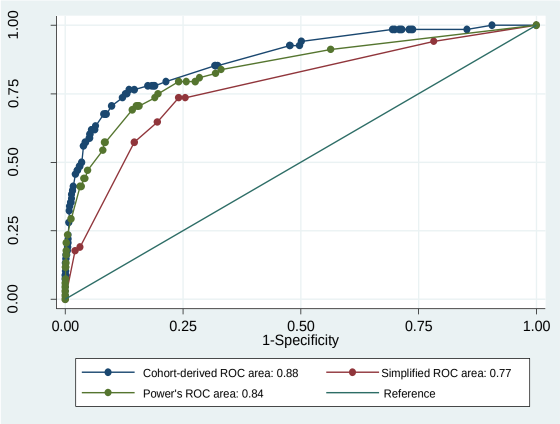

A study was conducted by KEMRI in 2011 to assess treatment practices for patients with STD and knowledge of symptoms compatible with AHI in Mtwapa and Shanzu, Coastal Kenya. This study surveyed practices at 23 health facilities and 20 pharmacies. One important finding was that although malaria transmission has reduced in recent years in Coastal Kenya,[24] presumptive treatment of febrile adult patients for malaria is common and diagnostic microscopy underutilized. Taken together, the 23 facilities reported having treated 250 patients for presumed malaria in the week prior to the survey, while the 20 pharmacies sold approximately 1000 malaria treatments in the week prior to the survey. In addition, compliance with treatment guidelines for urethral discharge was suboptimal, and HIV testing was not recommended to urethritis patients in the vast majority of cases, contrary to current recommendations.[25]

In the present study we want to assess if young adults who seek health care for symptoms compatible with AHI or an STD (defined as urethritis, proctitis, or general ulcer disease) can be targeted for AHI screening following an algorithm based on rapid HIV-1 tests for initial screening, p24 antigen testing at a central laboratory for seronegative and serodiscordant patients, then convalescent HIV-1 rapid tests repeated at community sites 2-4 weeks after initial presentation, for all seronegative or serodiscordant, p24-antigen-negative patients. If funding permits, we will evaluate the additional value of RNA testing for detection of AHI.

APPROVED
07 JAN 2013

5) Justification for the study

Identifying patients with AHI has received little attention in resource-limited settings, as low-cost, point-of-care RNA or antigen/antibody tests are not yet available for use in these areas. However, we have extensive experience using a low-cost p24 antigen assay that may perform well for this purpose. In addition, we feel that better implementation of rapid HIV-1 testing according to current recommendations, combined with intensive follow-up to ensure repeat testing, would significantly improve the chance to detect seroconversion. We therefore propose to evaluate whether identification of young adults and the AHI testing algorithm described above can identify AHI in up to 3% of patients seeking care at pharmacies or health centres. Those patients will benefit from learning their HIV-1 status and the opportunity to link into comprehensive care early if found infected. This study will also assess if pharmacies can be engaged to refer patients for HIV-1 testing, thus supporting strategies for the expansion of HIV-1 testing in Kenya.[26]

6). Null Hypotheses

1. Targeted screening for AHI among patients seeking health care for symptoms compatible with AHI or STD will identify very few or no AHI cases (<1% of those screened).
2. Intense follow-up of patients evaluated for AHI will not improve rates of repeat HIV-1 testing 2-4 weeks after health-care seeking, relative to standard practice (i.e., recommendation to return for testing on a given date).

7). **Objectives**

a. General Objective

To assess if young adult patients who seek urgent health care for fever and other symptoms compatible with AHI or STD can be identified, and undergo targeted screening for AHI.

b. Specific objectives

1) To assess current practice for the evaluation of Kenyan adults presenting with acute febrile illness, in terms of compliance with available guidelines and likelihood of detecting AHI.
2) To field test a symptom screening tool to identify adults seeking urgent care for symptoms compatible with AHI or an STD at pharmacies and health care facilities.

APPROVED
07 JAN 2013

3). To implement targeted AHI screening in 5 participating health care facilities by adding low-cost p24 antigen testing at presentation and repeat rapid testing 2-4 weeks later for all adults who are HIV-1-seronegative or serodiscordant at initial presentation.

4). To conduct a pilot randomized controlled trial (RCT) among 412 initially seronegative or serodiscordant patients to evaluate the efficacy of intensive follow-up vs. standard follow-up to promote repeat HIV-1 testing 2-4 weeks after initial presentation.

5). To assess acceptability of the testing algorithm among a sample of subjects, and the understanding of HIV-1 infection and risk behaviour among recently infected individuals.

8). Study design and methodology

(a) Study sites

The study will be carried out in the two adjacent coastal towns of Mtwapa and Shanzu, located in an area in which the KEMRI HIV/STI programme operates since 2005. Mtwapa and Shanzu each have a large number of bars, nightclubs, and hotels which facilitate tourism, sex work, and other businesses of a rapidly growing population. A recent survey enumerated, in the two areas, 57 health care providers (including 30 pharmacies), of which 23 clinics and 20 pharmacies participated in a survey of treatment practices for urethral discharge and understanding of AHI in 2011.[25]

For this study, we will expand our 2011 survey of health care providers in Mtwapa and Shanzu by selecting the 2 Government health centres and 6 randomly selected private health facilities of the facilities which participated in the KEMRI SSC 2029 survey for an audit of current practices (specific objective 1). We will then select 5 health facilities and 5 pharmacies from among those that participated in the recent survey of treatment practices for STD (KEMRI SSC 2029 , specific objective 2). To be eligible for specific objective 2, health care facilities must have at least one full-time staff member (nurse, clinical or medical officer, laboratory technologist) available for training, including VCT training (health facilities only) if not certified. Pharmacies must be willing to use the screening tool to assess young adults who ask to purchase ‘over-the-counter’ medication for fever, body pains, diarrhoea or STD, and to refer eligible patients to a participating health care facility.

(b). Study population

i. Criteria for inclusion:

APPROVED
07 JAN 2013

Any adult patient aged 18-29 years, who scores 2 or higher at a 'Symptom Screening Tool & study eligibility score list' [appendix 2]. As follows:

1). Self-reported fever, diarrhoea, or STD (each variable scores a 2); body pains, or report of multiple partners in past 2 months (each variable scores a 1). [pharmacies]

2). Confirmed fever (≥37.5 °C axillary), reported diarrhoea, or evidence of STD (variable score=2); reported body pains, or report of multiple partners in past 2 months (variable score=1) [health facilities].

Patients should be a resident in Mtwapa or Shanzu, or planning to stay in Mtwapa for approximately 4 weeks duration, willing to give locator information (including mobile phone number) and willing to undergo free evaluation for AHI (i.e., confidential l HIV-1 testing and counselling, p-24 antigen testing if rapid HIV-1 test results are negative or discordant, and convalescent HIV-1 testing at 2-4 weeks after first presentation).

ii. Criteria for exclusion:

Any adult patient who does not meet inclusion criteria as detailed above.

c) Sampling.

Hypothesis 1:

We assume that targeted screening of patients seeking health care for symptoms compatible for AHI or STD can identify AHI in 3% of patients meeting eligibility criteria. The expected number of patients and 95% confidence intervals given a point prevalence of 3.0% for different sample sizes is:

N	Patients with AHI	95% CI
100	3	0.6 – 8.5
200	6	1.1 – 6.4
300	9	1.4 – 5.6
400	12	1.6 – 5.2

A sample size of only 200 patients would suffice to reject the null hypothesis.

APPROVED
07 JAN 2013

Hypothesis 2:

We propose intensive follow-up in order to increase rates of repeat testing for HIV-1 after presentation to a health facility or pharmacy with symptoms compatible with AHI. With a convenience sample of 206 HIV-1-seronegative or serodiscordant patients (n=103 per arm) and an expected response rate of 50% in the intensive follow-up intervention group (n=52), and 30% in the standard-of-care group (n=31), we will have 80% power to detect a difference in study arms, given a 2-sided alpha= 0.05%. However, a study of 206 volunteers would only identify 6 (3% of 206) patients with AHI. As we aim to qualitatively assess the understanding of patients with AHI (objective 5) we wish to increase the sample to 412 HIV-1 negative or serodiscordant patients in order to identify 12 patients with AHI. *Appendix 3 for flow diagram.*

Note that our total sample will be 600. Of these, 80 will participate in the pilot phase (specific objective 2). With an estimated HIV-1 prevalence of 20% in young adults seeking urgent health care, 416 (520-[20%*520]) subjects will be HIV-1 negative or discordant, and randomised.

d). Procedures

<u>Specific objective 1:</u> We will (1) evaluate existing training curricula for nursing, clinical and medical officers in Kenya; (2) review national and international (WHO) guidelines for the management of febrile adult patients;[27, 28] and (3) asses current practices among a subset of health providers.

Audit of current practices: We will request the 'in-charge' from all Government health centers and 6 randomly selected private health facilities out of the 23 healthcare facilities included in the 2011 survey mentioned above to extract some limited clinical data of patients who presented with a fever. At each facility, after obtaining written informed consent, we will request staff to review clinic records of the first 12 adults presenting with fever in the past one week, extracting date of birth, gender, date of onset and duration of fever, additional symptoms reported, sexual risk assessment (if any), physical examination findings (including temperature), laboratory tests performed, working diagnosis, and treatment prescribed. A standardized data collection form will be developed and piloted before embarking on data collection.

APPROVED
07 JAN 2013

We will also request 5 pharmacies to keep an anonymous log of adult patients meeting eligibility criteria during a period of 2 weeks (appendix 4).

Study outcomes will include:

- Report on available guidelines and relevance to detection of AHI.
- Report on findings from the provider audit, including number and proportion of adults screened for sexual risk, number and proportion tested for HIV-1, number and proportion of those tested for HIV-1 in whom follow-up testing was recommended.
- Proposed algorithm for assessing patients with fever that integrates screening for AHI.
- Report on total number of adult patients meeting eligibility criteria identified at pharmacies over a two week period.

<u>Specific objective 2:</u> We will pilot a symptom screening tool (appendix 2) to identify adults seeking health care for fever, diarrhoea, body pains, or STD (defined as urethritis, proctitis, and general ulcer disease) at 5 health facilities and 5 pharmacies during two weeks in Mtwapa, providing close supervision and monitoring to determine the feasibility and acceptability of this screening tool in practice.

We will train staff from 5 health facilities and 5 pharmacies to use this new tool to assess approximately 80 patients who seek health care at pharmacies or health facilities in a pilot phase. After the training, we will observe implementation of the screening tool in practice. At each participating facility, approximately 8 patients with fever, diarrhoea, body pains, or STD will be invited to undergo HIV-1 testing. We will log each eligible patient who seeks health care at the 5 pharmacies and 5 health facilities during the pilot phase.

At the 5 pharmacies, patients who meet screening criteria will be referred to one of the 5 facilities with HIV-1 testing capacity. All referrals will be audited for appropriateness and tracked for completion, through a biometric-based system storing a unique alphanumeric number of the fingerprint scan of the referred patient. The stored study number (and not the fingerprint) is accessible only through special software maintained by investigators.

Study outcomes will include:

- Production of a written training manual and supervision guide.

APPROVED
07 JAN 2013

- Evaluation of the appropriateness of referrals and enrolments, and completion of referrals from pharmacies to participating health care facilities.

Specific objective 3: We will evaluate an additional 520 young adults seeking urgent health care for febrile illnesses, body pains, diarrhea, or STD at the 5 study-health facilities, with referrals from the 5 study-pharmacies. Patients will be screened and identified at pharmacies and health facilities. Pharmacies will refer patients to 1 of 5 health facilities but will not test patients for HIV-1 themselves. (see flow chart, appendix 3). Each eligible patient will be logged irrespective of their study interest. All eligible adults will be offered rapid HIV-1 testing (*Determine® and Unigold®*), malaria testing (*OptiMal® IT kit*) and targeted risk reduction counseling. Approximately 104 patients who test HIV-1 positive (assuming an HIV-1 prevalence of ~20%) will be referred to comprehensive care.

For all HIV-1-seronegative or serodiscordant patients, a 5-mL blood sample will be obtained for p24 antigen testing. These samples will be collected twice daily from each participating facility for testing at the KEMRI clinic in Mtwapa using the *miniVidas®* (Biomerieux, France; assay duration: 1.5 hours). Remaining sample will be stored for HIV-1 RNA testing, should funding permit. All p24-antigen-positive subjects will be traced within 24 hours of a positive test result by a community counselor, who will provide post-test and risk reduction counseling. Study outcomes will include:

- Number and proportion of patients targeted for AHI evaluation at pharmacies and health facilities.
- Number and proportion of patients identified at pharmacies and health facilities who enroll in the study
- Number and proportion of adults diagnosed with AHI by p24 antigen testing alone.

Specific objective 4: All patients with negative or discordant rapid HIV-1 test results on initial presentation will be invited for repeat rapid HIV-1 testing 2-4 weeks after the initial evaluation. For these patients, we will assess whether an intensive follow-up program including SMS reminders or phone calls and home visits for those who fail to return for testing will increase uptake of repeat HIV-1 testing 2-4 weeks after initial presentation. To achieve this objective, we will randomize approximately 412 HIV-1-seronegative adults to receive a standard appointment for repeat HIV-1 antibody testing at 2-4 weeks after initial testing, or an appointment with addition of a reminder mobile phone call or SMS if possible,

APPROVED
07 JAN 2013

plus a home visit by a community counselor if the participant fails to present on the appointed date.

Randomization. On average, each study health facility will enrol approximately 80 HIV-1 study subjects for AHI evaluation. As this number is likely to vary per facility, 'blocks' of 20 opaque envelopes will be provided to each of the 5 study health facilities, with replacement based on the number of people enrolled in the study. Each block will consist of 10 envelopes assigning the participant to standard follow up and 10 to intense follow up. Participants will be counseled concerning the randomization process. It will be explained that all patients with a positive p24 antigen test will be contacted by a study nurse within 24 hours after blood drawing, irrespective of their assignment. Participants will be asked to draw an envelope from the set of available envelopes at the facility. When fewer than 6 envelopes are remaining a new set of 20 opaque envelopes will be provided to the study site. Staff will record the assignment and verify locator and mobile phone number of the participant.

Study outcomes will include:

- Number and proportion of adults responding to standard appointment and number who responded to intense follow up to complete repeat testing 2-4 weeks after initial presentation.
- Number and proportion of adults diagnosed with AHI by rapid antibody seroconversion.

Specific objective 5: We will briefly interview 1 out of 4 patients screened regarding the acceptability of the screening process and their experience of the encounter during the pilot phase. We will use a semi-structured questionnaire exploring acceptability of screening and identification at pharmacies, referral to health centres and possible barriers to study participation and HIV-testing algorithm. Answers to questions will not be tape recorded, but notes will be taken by the counselor conducting the interview.

We estimate that 12 patients with AHI will be identified. We will assess each patient's transmission risk behavior and current life circumstances in order to offer tailored sexual risk reduction counseling at diagnosis, month 1 and 3 following diagnosis. Options for accessing comprehensive care will be discussed and partner counseling and -testing offered.

Participants will be asked to undergo a focused interview at the first visit after diagnosis. This baseline interview will use open questions to probe about the patient's understanding of

APPROVED
07 JAN 2013

safe sex and HIV, acute HIV infection and its diagnosis. Topics including the patient's knowledge of HIV transmission, self-reported risk behavior in the past several weeks, understanding of how to reduce risk, plans for disclosure, and understanding of care and treatment options will be explored. A follow-up interview at month 3 after diagnosis will use similar themes as the baseline interview. These personal interviews will be conducted by staff trained in qualitative research and will be recorded to enable detailed analysis.

Study outcomes will include:

- Summary report on acceptability of pharmacy referrals and HIV testing algorithm to clients, based on up to 20 interviews.
- Qualitative study of knowledge, behavior, and plans among newly diagnosed HIV-1 patients.
- Proportion of patients diagnosed with AHI who successfully enroll in HIV-1 care at 3 months post diagnosis.

9). Data Management

Date Storage. Except for informed consent forms, all data collection forms will carry unique alphanumeric identifiers and no patient names. Data will be stored in lockable cabinets in the data room of the KEMRI-clinic in Mtwapa which has restricted access. We use pre-printed & bar-coded stickers for data collection forms, laboratory request forms, and specimen identification. Laboratory samples are registered in an electronic Laboratory Information Management System (LIMS) at KEMRI (GCLP-accredited) in Kilifi. We have established p24 antigen testing capacity and will conduct daily p24 antigen testing –on samples collected by health facilities for the proposed study. P24 antigen results will be completed within 3 hours of sample collection on Monday to Friday when samples were collected before 2 pm, and next day morning completion before 12 am when samples were collected in afternoon or on weekend days. Data are entered and stored into a secure database, with restricted password access, and routine comprehensive backup.

APPROVED
07 JAN 2013

Staff at study pharmacies and health facilities will be trained and supervised by the principal investigators, who will also perform daily quality checks on collected data and provide feedback to study sites as necessary.

Data analysis plan. Data cleaning, recoding and analysis will be conducted using Stata® version 11 (StataCorp, College Station, Texas). The study is descriptive and will provide estimates of proportions of patients with AHI, and proportion of adults identified and screened, as has been outlined above. Exact binomial 95% confidence intervals will be provided for all proportions reported. Comparisons will be made between persons diagnosed with AHI and uninfected participants using Chi square or Fisher's exact tests for binary or categorical variables and the Mann Whitney U test or Student's t test will be used for continuous variables, as appropriate.

For the RCT of intensive versus standard follow-up, analysis will be modified intent-to-treat after exclusion of any p24-antigen-positive patients. The primary outcome will be completion of repeat HIV-1 serologic testing 2-4 weeks after the initial presentation. We will analyze the primary outcome using binomial logistic regression with generalized estimating equations to control for clustering by health care facility. In addition, we will compare baseline characteristics between the two groups (e.g., demographics, initial site of presentation), and if a statistically significant difference (i.e., an imbalance in a variable at baseline) is noted, then these variables will be tested to see if they also related to the outcome of interest. Any characteristic that is both unbalanced at baseline and related to the primary outcome will be include in final multivariable modeling. Crude and adjusted Odds ratios and 95% confidence intervals will be presented.

Qualitative interviews. Pilot phase interviews for acceptability of the screening and testing algorithm (n=20) will use Likert-scale and open-ended questions. Interviewers will write detailed notes after each interview, detailing their impressions and observations of the interview conversation. Opinions will be summarized. Pilot phase interviews will not be tape-recorded.

Recorded individual interviews (patients with AHI) will be transcribed verbatim into Swahili and then translated into English by professional transcription/translation staff who have experience in conducting this work for research purposes. Taped interviews will be transcribed by staff transcribers as soon after the date of interview as possible. To

APPROVED
07 JAN 2013

protect participants' confidentiality, transcribers will omit all personal identifying information from the transcripts.

Data from focused interviews with AHI participants will be analyzed using grounded theory approaches. Translated transcripts will be entered into NVivo, and analysis will aim to identify and categorize the attitudinal, psychosocial, and contextual factors associated with AHI. Data analysis will be iterative, including open coding, axial coding, marginal remarks, comparisons, and memo-writing. Themes will be analyzed and triangulated. The next stage of analysis will relate concepts in order to identify factors that can improve HIV prevention strategies, and enhance quality of HIV health services and counseling for AHI patients.

10). Time frame

The study is expected to start in January 2013 and be completed in December 2013.

a.	Audit	January 2013
b.	Pilot and Definite study	February – July 2013
c.	Data analysis	June – August 2013
d.	Report preparation	September - December 2013

11). Ethical Considerations

(a) Human subjects

i. Risks

For health providers who will participate, the study procedures may marginally increase the length of consultation per patient. The study will also involve asking questions about sexual behavior which may be uncomfortable for some of the health providers. We will ensure all participating providers give feedback on suitability of the interview tools. Training on interview techniques will be given where necessary. In addition, one staff from each participating health care facility will be offered training to qualify for HIV-1 testing and counseling. A reimbursement of KSh 100 per enrolled patient will be given for time spent on study procedures.

APPROVED
07 JAN 2013

For patients who will participate, blood sample taking may cause some discomfort or pain. In addition, the study procedures may take longer than a normal care visit, and will require an extra clinic visit for follow-up evaluation. Learning about HIV-1 infection may cause stress, and, if found infected, patients may feel isolated. Ksh 300,- will be given for time spent on study procedures and for transport to attend to the enrolment visit. We will not reimburse participants for their appointment visit 2-4 weeks later as the response to the appointment visit is the purpose of the study.

ii. Benefits to patients and community as a whole

Health providers who participate in the study will get an opportunity to improve their knowledge of AHI and skills in management of patients with febrile illnesses. Patients who participate will receive a free malaria test (when fever is present) and an HIV-1 test (all visits). HIV-1 infected patients who know their status may benefit from additional counseling and discussing care options available in Mtwapa and Shanzu. For patients who do not know their status, or have previously tested HIV-1 negative, learning their current HIV-1 status may be beneficial. Early diagnosis of HIV-1 will also enable the patient to take measures to avoid infecting their sexual partners and to register early into HIV-1 care. Some participants may feel altruistic about contributing to the development of a new targeted strategy to identify patients with acute HIV-1 infection.

Improvement of diagnostic practices for the evaluation of febrile patients is likely to benefit non-study patients who seek health care at the participating health facilities. Requiring malaria tests for febrile patients may help demonstrate that not all fevers are malaria. In addition, an improved approach to interviewing patients about sexual risk behaviors is expected to result in a reduction of risk among both HIV-infected and uninfected patients.

Individual informed consent process: Written informed consent will be obtained from all patients who will be enrolled into the study.

Community engagement strategy

In 2011, we engaged health care providers in the study area in a KEMRI survey of treatment practices for STDs. Before commencing this study, we will solicit feedback from selected health providers regarding potential feasibility and acceptability of the study procedures. Community engagement is a continuous process and KEMRI has

APPROVED
07 JAN 2013

established mechanisms to engage the general community in the study area, and health providers about the need to expand HIV-1 testing to patients seeking urgent health care. Upon completion of the study we will seek opportunities to report the results back to the health providers directly through feedback meetings and to national authorities through sharing of the results in conferences and seminars, as well as through scientific communications.

iii. Confidentiality

Confidentiality will be maintained in all phases of the study. Only number identifiers will be used and only summary statistics will be presented during dissemination. Strict measures will be taken to ensure that only the investigator team have access to primary data (e.g., by using passwords to protect electronic files and storing all completed questionnaires in lockable cabinets).

New Drugs: Not applicable

Animal subjects: Not applicable

12). Expected application of the results

The Kenyan Ministry of Health recommends that HIV-1 testing is provided to all patients seeking health care. This applies to patients seeking urgent care from pharmacies or health facilities. In practice, this is rarely done. This study aims to assess if targeted screening for HIV-1 can be helpful in identifying patients with acute HIV-1 infection. We anticipate that outcomes of this study will aid in improving HIV-1 testing guidelines for patients seeking urgent health care.

13). Budget

	Ksh	US $
Project staff	4,378,640	54,733
General administration, including 20% of Mtwapa clinic running costs	1,205,790	15,072
Patient travel and reimbursements	269,100	3,364
Equipment and laboratory costs	913,050	11,413
Education and training activities	506,250	6,328
Total	7,272,830	90,910

APPROVED
07 JAN 2013

Budget justification:

1. **Project staff.** Various project staff (pharmacist, study coordinator, tracer, clinician, community mobilisor) will be involved in the implementation of this project during an estimated duration of the study of 6 -9 months.

2. **Clinic running costs.** 20% of current clinic running costs (US $ 1675, per month) for 9 months. Costs include: connectivity, maintenance contract (i.e. air conditioners, lab equipment, generator, security of location), and maintenance of building.

3. **Patient travel and reimbursement.** Health facility data extraction & pharmacy anonymous log + travel reimbursement of staff to attend to feedback meeting (Ksh 500 * 20). Patient evaluation and enrolment (Ksh 600* 100). Patient referral (successful + coupon; Ksh 300*100 and Ksh 100 * 20), Patient reimbursement (first visit, Ksh 300*600). Personal interviews (Ksh 300*12).

4. **Equipment and lab costs.** Malaria optimal kits (n=500 – Ksh 270,000). P24ag test kits (n=450, Ksh 360,090). Rapid HIV tests (Determine and Unigold, Ksh. 308,013). Vacutainers, needles, gloves. Condoms, lubricants.

5. **Mobilisation and feedback.** Clinician training (on site and study start up). Printing of patient information sheet and coupon. Estimated: Ksh 506,250.

Appendices

1. Role of investigators *(CVs of Dr. Adrian Smith and Anisa Omar; provided as separate copies attached to this protocol)*

2. Draft symptom screening tool

3. Flow diagram

4. Anonymous pharmacy log file & health facility data extraction form; patient recruitment flyer; patient log and referral coupon - (*provided as separate copies attached to this protocol*):

   - Anonymous Pharmacy review log and Health facility data extraction form, version 1.4 – 19 December 2012
   - Patient information leaflet [English], version 1.4 – 19 December 2012
   - Patient information leaflet [Kiswahili], version 1.4 – 19 December 2012
   - Patient log and referral coupon, version 1.4 – 19 December 2012

5. Consent forms in English and Kiswahili *(provided as separate copies attached to this protocol)*
   - Informed Consent Document for health care facilities, version 1.4 – 19 December 2012
   - Informed Consent Document for pilot study, version 1.4 – 19 December 2012
   - Informed Consent Document for study proper, version 1.4 – 19 December 2012

APPROVED
07 JAN 2013

- Informed Consent Document for personal interview, version 1.4 – 19 December 2012
- Informed Consent Document for pilot study [Kiswahili], version 1.4 – 19 December 2012

- Informed Consent Document for study proper [Kiswahili], version. 1.4 – 19 December 2012

- Informed Consent Document for personal interview [Kiswahili], version 1.4 – 19 December 2012

APPROVED
07 JAN 2013

Appendix 1:

a). Role of investigators

Eduard Sanders: conception protocol, study design, protocol development, data analysis , manuscript writing

Henrieke Prins: project management, training of health providers, data collection, data management, and manuscript writing

Peter Mugo: study design, protocol development, project management, training of health providers, data collection, data management, and manuscript writing

Alexander Thiong'o: protocol development, training of health providers, sample collection, data collection, and manuscript writing

Elizabeth Wahome: data management and analysis

Elise van der Elst: study design, protocol development, manuscript writing

Adrian Smith: study design, protocol development, advice on data analysis and manuscript writing

Anisa Omar: advice on protocol implementation and community engagement

Susan Graham: study design, protocol development, expertise on clinical procedures, and manuscript writing

APPROVED
07 JAN 2013

Appendix 2: (draft) **Symptom Screening Tool & study eligibility score list**

1. Patient seeks treatment for fever — Please tick score:		
(health centre: fever >= 37.5 °C axillary). Yes=2	2	
	0	
2. Patient seeks treatment for a STD		
(urethritis, proctitis, or genital ulcer disease). Yes=2	2	
	0	
3. Patient seeks treatment for diarrhoea		
(3 or more loose or liquid stools per day, or more frequently than is normal for the individual). Yes=2	2	
	0	
4. Patient seeks treatment for generalized body pains		
Yes=1	1	
	0	
5. Patient reports more than one sexual partner in past 2 months		
Yes=1	1	
	0	
Patients' age is between 18-29 years? — Please confirm:		
Record: Date of birth __ (day)/___ (month)/____ (year)	Yes	
	No	
Does patient reside in the Mtwapa / Shanzu area?		
	Yes	
	No	
Is patient willing to take a confidential and free HIV-1 test?		
	Yes	
	No	

The eligibility score should be 2 or greater. Patient should be 18 years or older but <30 years, residing in Mtwapa or Shanzu and willing to take an HIV-1 test.

Always ask:

Has patient ever been tested for HIV? No__ Yes __

if Yes, date test __/__/____ (day/month/year) Result of last HIV-test: __ Neg; __ Pos; __ Unknown

If HIV was negative or unknown: recommend HIV-testing (PITC) and send to lab.

If patient not interested in study, reasons for refusal (tick one answer only):

Participant has not been sexually active in past 6 months	
Does not want to take an HIV-1 test	
Knows HIV-1 status and not interested in study	
Not interested in study [please ask for reason and capture under other]	
Other, pls describe:	

Form completed by __________________________ (name) Date __ / __ / ____

APPROVED
07 JAN 2013

Appendix 3: Flow diagram –**Targeted Screening of At-Risk Adults for Acute HIV-1 Infection**

Flow diagram does not include pilot phase (80 patients).

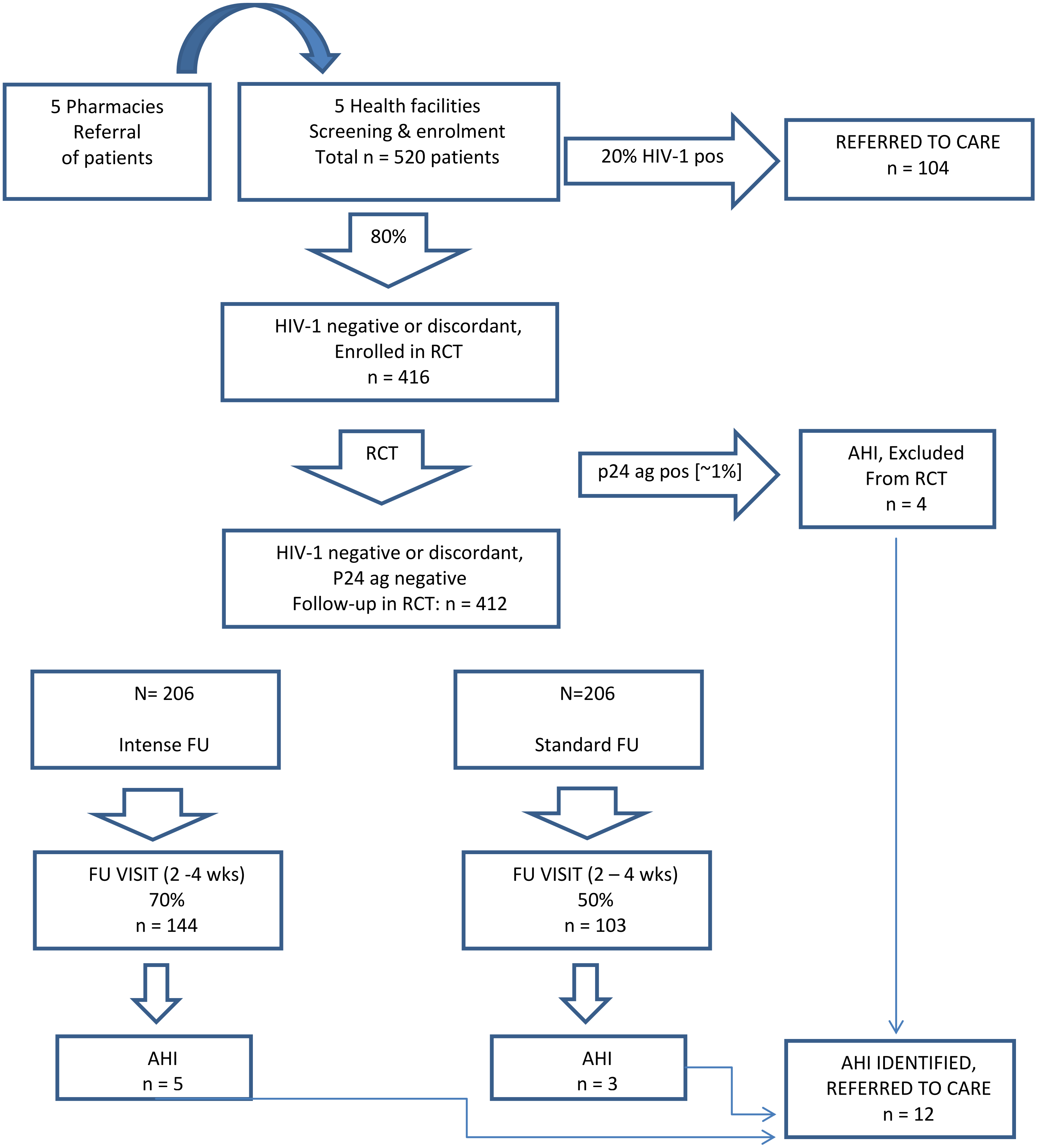

APPROVED
07 JAN 2013

References

1. Mwangi M, Bunnell R, Nyoka R, et al. Unsafe sex among HIV-infected adults in Kenya: results of a nationally representative survey. *Journal of acquired immune deficiency syndromes* 2011; **58**(1): 80-8.
2. Powers KA, Ghani AC, Miller WC, et al. The role of acute and early HIV infection in the spread of HIV and implications for transmission prevention strategies in Lilongwe, Malawi: a modelling study. *Lancet* 2011; **378**(9787): 256-68.
3. Pinkerton SD. Probability of HIV transmission during acute infection in Rakai, Uganda. *AIDS and behavior* 2008; **12**(5): 677-84.
4. NACC. Kenya Modes of HIV Transmission Study - Synthesis report. *Kenya National AIDS Control Council* 2008.
5. Tindall B, Barker S, Donovan B, et al. Characterization of the acute clinical illness associated with human immunodeficiency virus infection. *Archives of internal medicine* 1988; **148**(4): 945-9.
6. Schacker T, Collier AC, Hughes J, Shea T, Corey L. Clinical and epidemiologic features of primary HIV infection. *Ann Intern Med* 1996; **125**(4): 257-64.
7. Sanders EJ, Wahome E, Mwangome M, et al. Most adults seek urgent healthcare when acquiring HIV-1 and are frequently treated for malaria in coastal Kenya. *AIDS (London, England)* 2011; **25**(9): 1219-24.
8. Lindback S, Thorstensson R, Karlsson AC, et al. Diagnosis of primary HIV-1 infection and duration of follow-up after HIV exposure. Karolinska Institute Primary HIV Infection Study Group. *AIDS (London, England)* 2000; **14**(15): 2333-9.
9. Cooper DA, Gold J, Maclean P, et al. Acute AIDS retrovirus infection. Definition of a clinical illness associated with seroconversion. *Lancet* 1985; **1**(8428): 537-40.
10. Vanhems P, Allard R, Cooper DA, et al. Acute human immunodeficiency virus type 1 disease as a mononucleosis-like illness: is the diagnosis too restrictive? *Clin Infect Dis* 1997; **24**(5): 965-70.
11. Pilcher CD, Christopoulos KA, Golden M. Public health rationale for rapid nucleic acid or p24 antigen tests for HIV. *The Journal of infectious diseases* 2010; **201 Suppl 1**: S7-15.
12. Powers KA, Miller WC, Pilcher CD, et al. Improved detection of acute HIV-1 infection in sub-Saharan Africa: development of a risk score algorithm. *AIDS (London, England)* 2007; **21**(16): 2237-42.
13. Bassett IV, Chetty S, Giddy J, et al. Screening for acute HIV infection in South Africa: finding acute and chronic disease. *HIV medicine* 2011; **12**(1): 46-53.
14. Cohen MS, Gay CL, Busch MP, Hecht FM. The detection of acute HIV infection. *The Journal of infectious diseases* 2010; **202 Suppl 2**: S270-7.
15. Fiebig EW, Wright DJ, Rawal BD, et al. Dynamics of HIV viremia and antibody seroconversion in plasma donors: implications for diagnosis and staging of primary HIV infection. *AIDS (London, England)* 2003; **17**(13): 1871-9.
16. Wolpaw BJ, Mathews C, Chopra M, Hardie D, Lurie MN, Jennings K. Diagnosis and counselling of patients with acute HIV infection in South Africa. *Sexually transmitted infections* 2011; **87**(1): 71-2.
17. Fiscus SA, Pilcher CD, Miller WC, et al. Rapid, real-time detection of acute HIV infection in patients in Africa. *The Journal of infectious diseases* 2007; **195**(3): 416-24.
18. Lavreys L, Thompson ML, Martin HL, Jr., et al. Primary human immunodeficiency virus type 1 infection: clinical manifestations among women in Mombasa, Kenya. *Clin Infect Dis* 2000; **30**(3): 486-90.
19. Bebell LM, Pilcher CD, Dorsey G, et al. Acute HIV-1 infection is highly prevalent in Ugandan adults with suspected malaria. *AIDS (London, England)* 2010; **24**(12): 1945-52.
20. Serna-Bolea C, Munoz J, Almeida JM, et al. High prevalence of symptomatic acute HIV infection in an outpatient ward in southern Mozambique: identification and follow-up. *AIDS (London, England)* 2010; **24**(4): 603-8.
21. Braunstein SL, van de Wijgert JH, Nash D. HIV incidence in sub-Saharan Africa: a review of available data with implications for surveillance and prevention planning. *AIDS Rev* 2009; **11**(3): 140-56.

APPROVED
07 JAN 2013

22. Sanders EJ, Wahome E, Okuku HO, Mugo P, Thiongo A, Graham SM. When men may acquire HIV-1... Evaluation of a risk score vs. a simplified algortihm for detection of acute HIV-1 infection in men in Coastal Kenya. *Reviews in Antiviral Therapy & Infectious Diseases* 2012; **4**: 26-7.
23. Sanders EJ, Wahome E, Mwangome M, et al. Most adults seek urgent healthcare when acquiring HIV-1 and are frequently treated for malaria in coastal Kenya. *AIDS (London, England)* 2011.
24. O'Meara WP, Bejon P, Mwangi TW, et al. Effect of a fall in malaria transmission on morbidity and mortality in Kilifi, Kenya. *Lancet* 2008; **372**(9649): 1555-62.
25. Mugo PM, Duncan S, Mwaniki SW, et al. Treatment practices for urethral discharge among private and public health providers in coastal Kenya: opportunities for HIV prevention. *Reviews in Antiviral Therapy & Infectious Diseases* 2012; **4**: 27-8.
26. NASCOP. National Guidelines for HIV Testing and Counselling in Kenya. Nairobi: Ministry of Public Health and Sanitation 2010; 2010.
27. WHO. Guidelines for treatment of malaria - 2nd Edition. *World Health Organization, Geneva, Switserland* 2010; (ISBN 978 92 4 154792 5): 1-195.
28. WHO. Acute care - intergrated management of adolsecent and adult illness. *World Health Organization, Geneva, Switserland* 2009.

APPROVED
07 JAN 2013
